# Anti-colonial thought and global social theory

**DOI:** 10.3389/fsoc.2023.1143776

**Published:** 2023-03-30

**Authors:** Sujata Patel

**Affiliations:** Independent Researcher, Pune, Maharashtra, India

**Keywords:** anti-colonial thought, global social theory, post-colonialism, coloniality and decoloniality, subalternity, extraversion, indigeneity and endogeneity

## Abstract

From the late 1980s onward, global social theory has been introduced to a new perspective variously called indigeneity, endogeneity, Orientalism, Eurocentrism, post-colonial, decolonial, and Southern sociology/social sciences. This study argues that the above-mentioned trends should be collectively termed anti-colonial social theory as all of these explore the relationship between colonialism and knowledge production. The study divides the growth of anti-colonial social theory in terms of two phases and relates it to changing geopolitics of the 20th century. It argues that these distinct trends manifest a united stance in its ontological-epistemic articulation. It also argues that anti-colonial social theory can play a relevant role in a knowledge system divided through colonial/imperial relationships, given its theorization on the same.

## Introduction

Though anti-colonial thought and action have existed for many centuries, a comparative global discussion on “anti-colonialism” in the English scholarly language is only a few decades old (Abernethy, 2000; Bayly, 2004) and deliberation on anti-colonial social theory is even more recent (Go and Watson, 2019; Go, 2022). The word *colonialism* does not appear in English dictionaries until the mid-19th century though it has had a long history; it is now acknowledged that colonialism was initiated in the late 15th century and continues today in various guises. Osterhammel (2005) identified three modular types of colonialism: Colonies of exploitation where the primary goal was to exploit raw materials, land, and labor (e.g., British India); colonies of settlement (e.g., Spanish and British America and parts of Africa) and territories of strategic military significance (Portuguese control of the Indian Ocean or the United States in the Caribbean). As colonialism progressed and spread across various regions of the world and as different national European empires affected their domination globally, the above modular forms got mixed in the actual experience of power and control. At its peak, major European countries, together with the United States, colonized most of the Americas, Africa, Oceania, and Asia. From the 19th century, the Japanese empire also embarked on a similar process through the colonization of Korea. It is suggested that there are many other examples of contemporary colonization, such as Palestine.^1^

The first theorizations on the relationship between capitalism and colonialism were presented by Karl Marx^2^; this was later extended by Lenin and other Marxists. Thus, research and analysis of colonialism and anti-colonial thought most often follow a Marxist intellectual legacy (Gandhi, 1998).^3^ Early scholars have tended to distinguish colonialism from imperialism, and both processes use similar forms of domination and control. In colonialism, a metropole state controls territories through military interventions and uses violence, law, and policies to dominate peoples and territories beyond their own boundaries, while in imperialism, this domination occurs without the control of territories and through exploitative and exclusionary economic and political influences (Osterhammel, 2005). After the formal demise of colonialism, most scholars have used these two terms interchangeably, while some others term the contemporary processes of domination and control as neo-colonialism (Langan, 2017).^4^

Anti-colonial thought, in its many versions, maps and interprets ideas and actions that have emerged in the political struggle(s) of the colonized peoples against capitalist colonialism's material exploitation, ideologies, and practices. It collates, catalogs, and analyses the subjective experiences of being dominated by colonial and imperial economic, social, political, and cultural institutions, policies, and rules (Ashcroft et al., 2007, p. 11–12). While anti-colonialism has been described as a set of events, a historical process, and a series of social movements against it, punctuated through overt and covert violence on the subjugated population, anti-colonial thought is the analysis of the voices of the subjects as articulated and represented in prose, poetry, art, and within its aesthetics. It is an analytical and philosophical system of ideas, which combines ideas derived from Western religious, philosophical, and political sources merged with pre-colonial cultural and philosophical stances and framed in terms of the subjective experiences of those who were colonized. It uses this assessment to present ways to analyze the nature of metropolitan domination and its impact on the colonized societies and its various groups using proto-sociological positions to comprehend the social hierarchies that colonialism has constituted in colonized territories while simultaneously outlining the political strategies needed for confronting the military might and the violence perpetuated by the colonial authorities (Elam, 2017).

Anti-colonial thought has a long history of over 400 years and has evolved over time in the context of global colonial geopolitics. During this long history, it has changed and reorganized its ideas and ideologies and analytical stances. In addition to time, spatial issues have determined its content.^5^ As a body of thought, it has been regionally specific^6^ and geographically diverse, articulating specific colonial processes that have exploited the peoples of certain territories. These present forms and facets of exploitation and subordination in different kinds of capitalist colonialism by different powers. They highlight how these have created miseries and committed genocide of the peoples and their cultures at different times and in diverse ways. Such differentiation is true even within single colonies where colonial/imperial domination has been subject to the vicissitudes of uneven capitalist development (Parry, 1987).

All anti-colonial perspectives^7^ share the following characteristics: a search for a method to debunk received ideas, principles, and assumptions that naturalize colonial domination within the colony and in the mother countries. These help in designing a new perspective of theoretical analysis and method and delineating knowledge from the colonized point of view. As mentioned earlier, this new perspective could draw its intellectual resources from dissenting western positions and combine these with pre-colonial thought in its search for a new analytical stance. In this *avatar*, anti-colonial thought presents itself as an independent and sovereign perspective that can interrogate the logic of colonial domination and subordination. Second, it assumes that colonialism is a historical watershed and a marker of capitalist exploitation of its peoples, regions, and territories. Consequently, many anti-colonial organic intellectuals' and thinkers comprehend the past of their regions as a golden age that occurred before the advent of colonialism and use it to design a new utopia outside colonialism for its future. Finally, anti-colonial thought searches for critical epistemic voice(s) that can represent the subjective experiences of being exploited in order to comprehend the consequences of colonialism analytically.^8^ A political economy approach becomes part and parcel of anti-colonial thought.

From the above, it is clear that anti-colonial thought is a proto-sociological analysis of the roles and interventions of “native” groups for or against colonialism. It assesses in various ways the constitution of hierarchies and domination/hegemony in colonial territories.^9^ Anti-colonial thought takes the first step toward building an epistemic and methodological exercise that uses reason and rationality to search for a new logic to comprehend the relationship between power and knowledge under colonialism/imperialism. Together with the ideas of what constitutes a good society, anti-colonial thought offers an ontological-epistemological and moral perspective to examine and evaluate colonial capitalism. Through this proto-sociological approach, it contributes to the formation of social theory, a set of methodologies to deconstruct fields and disciplines legitimized within contemporary colonial/imperial geopolitics. This study elaborates on how anti-colonial social theory in its various versions emerged in the 1940s within the colonized regions of the world and found a presence in the 1980s within North American and European academia.

## Setting up the discussion: On anti-colonial social theory

Anti-colonial thought found its first theoretical expression as a social theory in the late 1970s through post-colonial thinking.^10^ It emerged approximately four decades back within the fields of comparative literature and humanities with the publication of Edward Said's *Orientalism* (Said, 1978) and later of *Culture and Imperialism* (Said, 1993). This new perspective which was fashioned by the scholarship of Edward Said, Homi Bhabha, and Gayatri Chakravorty Spivak—these three being called the theoretical trinity of post-colonial studies—has since found legitimacy as a mode of doing critical theory within academies of North America and Europe and has increasing spread in this *avatar* across the world (Schwarz and Ray, 2005; Albrecht, 2020). With the decolonial position^11^ (Mignolo, 2011; Mignolo and Walsh, 2018), a perspective promoted in the late 1990s by the USA based Latin American Studies programme, they have stamped ways to rethink the analysis of literature and lives of the colonized in all parts of the world. Increasingly, the post-colonial and decolonial gaze has been focused on analyzing migrants and/or the diasporic communities within the heart of the Empire. Today post-colonialism and decoloniality are used extensively to comprehend “otherness”; it expresses itself in the analysis of gender and race and also in domains, such as “queer and ecological projects” (Tlostanova, 2012, p. 130).

Thus, it is no surprise that since the beginning of the 20th century, post-colonial thought has found resonance within the field of sociology in North America and Europe; two critical texts of Bhambra (2007) and Go (2016) framed this intervention. These texts use a post-colonial perspective to examine the impact of the colonial episteme on the organization of sociology within the United Kingdom and the United States. These interventions follow the opening up of the intellectual space that occurred due to the breakdown of sociology's late 19th century positivist perspective, which assessed regularities, made law-like analyses, and used regression-based variable models to comprehend the “social.” While some scholars, in the 70s and the 80s, took the route of applying hermeneutics, interpretative, and constructivist analysis, some others suggested a need to historicize the discipline in order to comprehend whether the sociological classics and its cannons have relevance in comprehending new modernities being constituted within Europe (Lash, 1999).

Connell's (1997) discussion on this theme shifted and overturned the debate, which was until then restricted to an assessment of the relevance of the cannons in order to comprehend contemporary modernity in Europe. She queried the relevance of the classics given their moorings in the Empire's colonial and imperial projects. In many ways, Connell resonated with the argument and discussions inaugurated by the Marxist political economist Samir Amin in his book *Eurocentrism* (Amin, 1989). In this text, Amin argued that Eurocentrism was the dominant cultural, intellectual, and ideological representation of Western capitalism. Europe's intellectuals and social scientists claimed that Europe's history was unique because it incorporated a set of progressive ideas drawn from Greek and Roman civilizations, which were then incorporated into the Enlightenment. Amin argued that this claim needs to be interrogated through evidence-based historical analysis. Amin's argument found resonance with the publication of Bernal's *Black Athena* (Bernal, 1987), which contended that the Greek knowledge systems and their civilizing traditions were not unique to Greece. Rather these were heavily borrowed from the Egyptians and the Phoenicians. If these two books debunked the idea of Greece, and later Rome being the source of the exclusive nature of European progressive thought. Thus far, the origin of its social sciences was the publication of *Open the Social Sciences. The Report of the Gulbekenian Commission on the Restructuring of Social Sciences* (Wallerstein, 1996, 1997) marked an important milestone in this debate. This text is about the growth of social sciences in Europe and its institutionalization within the University system in the late 19th century during capitalism's imperialist phase. It used the historical approach to critically examine how Eurocentric assumptions of linearity and dualities structured this growth and legitimized a European understanding of social sciences. It argued that the divisions in its methodological approaches based on a notion of science directed the classification of knowledge into various disciplines of study and, in turn, steered the adoption of specific theories and methods used within these fields.

However, it was the publication of Raewyn Connell's text *Southern Theory* (Connell, 2007) that marked a watershed in understanding the relationship between colonialism and social sciences. While the discussions mentioned above examine the impact of colonial and imperial geopolitics on European social sciences, *Southern Theory*^12^ debated the sociological scholarships in Nigeria, Iran, India, and Latin America to contend that these should have equal epistemic valence with those from Europe and North America. Connell, like De Souza Santos (2014) after her, used the term *South* as a metaphor. Resonating post-colonial thinkers and Marxists mentioned earlier both argue that power is central to knowledge formation. Particularly, Connell contends that the discipline of sociology alludes to “authority, exclusion, and inclusion, hegemony, partnership, sponsorship and appropriation-between intellectuals and institutions in the metropole and those in the periphery” (Connell, 2007, p. ix). For Santos and Connell, “the South” was not a geographical territory but a knowledge periphery or a consciousness of a social sector of the population (e.g., the subalterns) within certain geo-political areas of the planet. Though there has been criticism of Connell's use of the South as a generic category (Emirbayer, 2013), she uses this concept as an entry point to map anti-colonial social thought.

This study follows the trail laid down by Connell when she argued that anti-colonial social theory could fill an intellectual space left open by the crisis of positivism that attacked the discipline in the 70s. It also argues that using anti-colonial as a generic term acknowledges its genesis in anti-colonial political struggles across the world and binds the different versions with an epistemic search for creating knowledge about a new society through a critique of the colonial. It is an umbrella term to describe a range of different methodological positions that have emerged in the wake of colonialism: indigenous sociology, indigeneity, and indigenous methodology (Atal, 1981; Akiwowo, 1986, 1999; Smith, 1999; Odora, 2002); endogeneity and endogenous thought; extraversion (Hountondji, 1995, 1997, 2009); autonomous and independent sociologies (Alatas, 2006); subaltern theory, derivative nationalism, and colonial difference (Guha, 1982; Chatterjee, 1986); colonial modernity (Barlow, 2012; Patel, 2017); internal colonialism (Martin, 2018); coloniality of power (Quijano, 2000); border thinking and de-linking (Mignolo, 2007); connected sociologies (Bhambra, 2014a); and post-colonial sociology (Go, 2016), south theories (Fiddian-Qasmiyeh and Daley, 2018). Undoubtedly these different positions highlight unique attributes, but they also flag an imperative for a common denominator that binds these perspectives. I suggest that this common denominator is the affiliation of these approaches to an anti-colonial social theory as an ontological–epistemological perspective.^13^

This study contends that anti-colonial theory is a sociologically grounded philosophical reflection on and about meta-theories in the context of colonial capitalism. It combines a methodology that debunks the use of dominant/hegemonic forms of logic and reasoning while searching for an original ontology that comprehends innovative ways of knowing and thinking to comprehend colonialism's exploitative and exclusionary processes constituting inequalities within the world. Anti-colonial theory, this study argues, presents us with a novel way of doing social sciences; it is the methodology of how to theorize rather than an elaboration of what it is. In this perception, it constantly interrogates sociologically the empirical, the theoretical, and the “scientific unconscious”^14^ that organizes fields/disciplines to present a new alternative (Rutzou, 2018).

Today, a space has been created for the existence of the “normative” within sociology (Chernillo and Raza, 2022). In this space, this study argues, anti-colonial social theory can be seen as one intervention. Consequently, it suggests that the first task is to map the methodological arguments presented by scholars divided by circuits of knowledge geographies and bring these into conversations with each other. It argues that it is possible to discern the origin of anti-colonial social theory in the late 1940s within the growth of anti-colonial movements in South Asia, Africa, and Latin America. Here, scholars started using anti-colonial thought as a site to conceptualize a new social science for the colonized territories and use it for constituting social sciences for emerging nation-states. The study argues that anti-colonial social theory postulates not only methodologies for deconstructing dominant/hegemonic positions across various geographies but also lays out steps to reconstruct them in new and novel ways in the context of global divisions of knowledge. These have many *avatars*, and the goal is to bring into conversation the distinct approaches while highlighting the commonality in terms of the ontological epistemic. These approaches share a fundamental question: what pathways of knowledge-making need to be followed to constitute new ones? If read together, do these present the methodological designs that have been developed to interrogate colonial social sciences? What alternatives do these offer for refarming colonial/imperialist thought?

In order to comprehend these tools and methodological apparatuses, this study outlines the evolution of these meta-theoretical ideas in anti-colonial social theory through two phases of their growth, from the 40s to 80s and from the 80s to the present, across various knowledge geographies. It also highlights the internal critiques that have developed within this scholarship as scholars after interrogating their legacies have redesigned tools to comprehend the organic links between traditions within disciplines and their relationship with power. In the last section, the article outlines the themes that this mapping raises and asks to elaborate on the constraints that exist in developing anti-colonial social theory as a global theory.

## The first phase: 1940s–1980s

The proto-history of the theories of colonial modernity lies in the interventions made by organic intellectuals in the colonized regions that initiated a re-conceptualization of late 19th-century economic and social theory. Jose Rizal (1861–1896) from the Philippines highlighted the civilizational character of the Philippine pre-colonial society and criticized Spanish colonial knowledge that blamed contemporary backwardness on the indolence of the Pilipino peasantry. His studies argued that the exploitation and extraction of resources by colonial Spain made the Philippines backward rather than its peasantry's indolence, as suggested by the Spanish (Alatas, 2010). Again, in the late 19th century, India's Dadabhai Naoroji (1875–1917) presented the drain theory that combined three arguments of colonial exploitation: How England's political control of India allowed it to transfer India's wealth to itself and, in turn, expand British economy and it colonial footprints; how it deindustrialized India and destroyed its textile industry in order to ensure that Indian textiles did not compete with English textiles, and finally, how it created fiscal policies that made Indians pay for the governance of this extraction, thereby exploiting the country twice over (Chandra, 2010).^15^

These early forays consolidated themselves in the 1940s and divided themselves into liberal and Marxist perspectives on colonial knowledge construction. Both asserted the necessity for colonial countries to have intellectual sovereignty/self-rule in knowledge production. Intellectual investments were made in liberal and Marxist economic and political theory, both demanding a new model of economic and political development-that of “autarky,” an import substitution industrialization. The liberal perspective was consolidated as a dominant position when ex-colonial countries from Asia and Africa, now networked into the non-aligned group post-Bandung, followed each other in investing in intellectual infrastructure institutions of teaching, research, and publication for supporting human resources that could frame autonomous social sciences in order to create these models outside the influence of capitalist and communist designs. Soon this network formulated a theoretical and methodological perspective termed indigenous or indigeneity that discussed the methods and methodologies that can produce relevant social science knowledge by scholars who were not made “captive” in their “minds” by colonialism.^16^

Over the course of the next decades, scholars converted this need for a sovereign stance into what is called the “indigenous perspective.” This perspective uses a combination of the methods, such as use of local sources and local languages; need for research by natives/citizens/insiders; alignment with nationalist agendas; and engagement with local philosophical and cultural traditions to constitute the space for alternate ways of doing social sciences (Atal, 1981). Over time, in most African and Asian countries, the indigenous perspective in economics and political sciences dovetailed with modern liberal nationalist and policy agendas, while sociology and anthropology valorized native cultures.^17^ However, outside this nationalist liberal policy orientation, an effort to sustain a critical perspective was made through Marxist positions. However, by the late 70s and 80s, the decrease in the influence of the left and Marxist positions, including that of official communism together with the slow decline of the non-aligned/third world movements and the incorporation of nationalist liberal concerns with the neoliberal theories promoted by the global North, caused a slow death for the quest of both liberal and Marxist indigenous social sciences (Patel, 2021a).

In the 80s and 90s, when the new bipolar world order came to be constituted, a renewed effort to create a critical anti-colonial social science emerged once again. In the wake of the demise of modernization theories and the slow decline of positivist methodology, these interventions have combined Marxist historical scholarship with structuralist, post-structuralist, and deconstructive positions to present, once again, tools to critically re-examine colonial/imperial knowledge and the nature of modernity in the ex-colonialized countries and thereby to reframe global social theory (Patel, 2021a,b). Contemporary interventions have critiqued current dominant/hegemonic perspectives in social sciences while examining their epistemic moorings not only in colonialism/imperialism but also within nationalist movements and in the nation-state's political projects. Below, I briefly narrate the concerns structuring five distinctive trends^18^ of social theory in this domain.

## The second phase: 1980s until today

First, in North and West Africa, there developed a Marxist perspective to re-orient the received indigenous approach.^19^ Initially, scholars asked questions regarding the limitations of the indigenous perspective in terms of its methods of study: Can one generalize from local sources and local languages that are restricted to ethnic groups across other groups within the nation-state, the region, and the continent? And are folk songs, myths and/or other oral traditions adequate representations of contemporary culture(s)? Can these sources frame the constitution of sociological theories? Soon, there developed a substantive critique of indigenous thought. Developed by the Benin philosopher Hountondji (2009), it argued that the term “indigenous” had colonial moorings and was conceptualized within European African Studies programs and assumed European methods of studying the “other.” This perspective, according to Hountondji, was ideological because it was objectified and promoted a partial understanding of the “other.” He thus argued that using local resources and local languages or native/insider research or a search for philosophical traditions based on ethnic cultures can yield good practices for constituting autonomous and scientific social sciences of Africa. Scholarship in Africa, Hountondji^20^ contended, needs a new perspective which he termed endogenous.

This approach did not reject the use of local or native-insider knowledge but suggested a need to include methodological protocols that helped to identify and recognize oral traditions that may have relevance. These, Hountondji contended, need to be interrogated in systematic scientific ways such that these can present new hypotheses or a theory. Hountondji argues that scholars should first assess the processes that reorganized and erased the many memories of cognitive thought in Africa, such as forced migration and slavery, before identifying such sources for investigation. He argued that local sources might have been overlaid by the objectification made by colonial authorities in distinct historical times, and if accepted, these would become ideological. He also maintained that critical scientific rationalities had not developed within African knowledge traditions to interrogate such local knowledge and thus comprehend its contemporary relevance. Without these, it would be difficult to separate the ideological from the scientific. In these circumstances, a search for pre-colonial local knowledge and cognitive traditions can only be possible, if at all, within marginal cognitive traditions whose practices were not tainted by colonial knowledge. Hountondji argued that unearthing these and interrogating them with scientific rationalities is particularly important in today's context-the late 1990s, given that contemporary African knowledge fields were characterized by academic dependencies, which he called “extraversion” (externally produced knowledge). The latter was defined as a process by which knowledge fields are circulated and reproduced in the periphery of the metropole as academic tourist circuits.

As against the above, another pathway, the second, was established with the publication of a series of 12 edited volumes in the 80s to 90s in the field of history by the Subaltern studies group. These volumes argued that Indian liberal and Marxist historiographies were rooted in nationalist-indigenous thought and thus reproduced bourgeois colonial categories. The Subaltern scholars presented to the world a new methodology to deconstruct colonial archival documents in order to comprehend the dominant/hegemonic colonial and nationalist knowledge. As a first step, they suggested the need to critique liberal and Marxist historiographies that highlighted the politics of the British-educated middle-class interest-group politics and projected its nationalism as ant-colonial. Instead, they argued that this group of elites, together with the other groups such as landlords/moneylenders and businessmen, wanted a share of political power in the colonial state and thus colluded with the colonial state.

Subaltern scholarship termed Indian nationalist historiography elitist because it did not analyze the anti-colonial protests of the “subalterns”—groups of peasants, tribes, and informalized workers who rebelled and resisted the colonial authorities but were made invisible in history writing. They contended that liberal and Marxist historians argued that these groups were pre-political and that because their resistance was not articulated in class terms nor manifested class solidarities, these were not actors who defined anti-colonial movements. As against this, Guha (1982) and his colleagues presenting the subaltern argument contended that the protests by these subalterns were indeed political. They argued that though their voices expressed non-class consciousness, this does not imply that they were voicing pre-colonial-capitalist primordial identities. Subsequently, subaltern scholarship has analyzed the various fragmented expressions articulated by the subalterns in and through religious idioms, songs, and ballads. It has assessed how their community identities of kin/subcaste/caste or tribal groups have helped the subalterns forge new identities. This scholarship has presented a new methodology for interrogating archival documents and has mined popular beliefs, folklore, and rumors to explain the political logic of such religious-political assertions during colonial and post-colonial/nationalist times.^21^ This scholarship continues the tradition of the Gramscian Marxist legacy that took credence to the significant role played by the peasantry in expressing a new epistemic voice against landlord exploitation.

One arm of subaltern scholarship also found resonance with post-colonialism enunciated by a group of West and South Asian migrant scholars in the United States who made contributions to the field of Comparative English Literature. The latter contended that during the 18^th^ and 19th centuries, the West had created and consumed an imaginary Orient to perpetuate its discursive power through literature and language (Said, 1978). This position, the third one, asserted that Orientalism was not only a field of knowledge or a discipline, or a set of institutions, or a corporate institution that primarily studied oriental societies and their cultures within Western universities, but rather it was a mode of thought based on a particular epistemology and an ontology that divided the Orient from the Occident. Following Foucault, post-colonialism declared that knowledge–power combined to produce its objects of study as a discourse and that this episteme resisted change and transformation because of its linguistic constitution. Subsequently, in this scholarship, there were no phenomena outside of language; thus, language, according to the post-colonialists, defines the character of Orientalism and produces the Orient as an object of knowledge, and establishes its outcome in terms of the relations of power. Post-colonial studies, in this sense, is a radical methodology that questions both the past and the ongoing legacies of European colonialism to undo them by interrogating its epistemic authority based on institutional power. Scholars using post-colonialism have redefined politics as critiquing fields of knowledge, as “theoretical practice,” a methodology that can transform relations of power within knowledge through its deconstruction. A section of the subaltern scholars affiliated themselves with post-colonialism. It argued for a radical critique of the discipline of history, given its dependence on the archives as a method of documenting the processes of change. Thus, if literature/language is the site of power for literary scholars, the colonial archive is the site of colonial power and authority for these post-colonial subaltern historians.^22^

The fourth pathway was developed with the enunciation by Quijano (2000), a Peruvian sociologist of the concept of coloniality.^23^ This shifted the discussion to an assessment of the colonial experience in Latin America. The concept of coloniality/coloniality of power combines, in new and radical ways, the mid-20th century project of conceiving an ontological epistemology of colonialism by integrating it with alternate political modernity. Quijano follows up the work presented by dependency theorists, world system schools, early Latin American historians, and the contemporary Marxist scholar Samir Amin to argue for the importance of querying Eurocentric assumptions regarding modernity to map its alternative(s). He constructs his argument through the Marxist historical, sociological method, as he maps the growth of colonialism through the trade circuits constituted by Iberian capitalism with the Americas from the 15th century and integrated later with Africa at the beginning of slavery in the 16th century. For Quijano, this model of colonial capitalism-land appropriation, resource extraction, and in migration was first institutionalized in the Americas. Over time, he argued, it had become a global cognitive model. The latter, he contends, was reproduced simultaneously in Europe and Latin America in the form of Eurocentrism, whose assumptions are “a peculiar dualist/evolutionist historical perspective” (Quijano, 2000, p. 556).

Quijano suggests that these assumptions of Eurocentrism formed the basis of the European scientific–technological development during the 18th/19th centuries and were imbricated in many other theories of universal history and culture, which influenced the formation of the social sciences in the late 19th century. Gradually, Quijano argued, it formed the contours of an ideology and of a diffuse common sense that has seduced differently the distinct and varied populous of the world it has encountered in covert and overt ways. Quijano also highlights the need to know how power embeds itself within scientific knowledge. While he contends that it is necessary to develop methodologies to unravel the consequences of what has occurred, e.g., his theory of coloniality which examines how values and norms are institutionalized in everyday life, within the family system and marriage alliances, within sexualities, in education, its pedagogies and its philosophies, it is also necessary to continuously deconstruct its relationship with the making of knowledge because methodologies also may become embedded in ideological positions and can serve dominant interests. Consequently, both theories and methodologies need constant scientific interrogation, even if these have emancipatory origins.^24^

The fifth trend, decoloniality, emerged within the Latin American Studies Programme in the United States. It draws on Quijano's concept of coloniality of power to present a worldwide perspective that argues that the discursive circle of colonial/capitalist modernity, of the duality/binaries of “I” and the “Other,” was initiated in the 16th century and this form spread itself around the world. It came to be institutionalized when the twin processes of the expulsion of Jews and Muslims from Spain and the elevation of Western Christianity to religious dominance led to an early racial classification. Following Quijano, decoloniality theorists argue that the American continent became the first contact zone and battleground for deploying Eurocentric ideas of civilization, evangelization, empire, racial difference, and subalternation of the knowledge of the colonized. It presents a repertoire of methodological concepts, such as Occidentalism (the formation of specific forms of racialized and gendered Western selves as the effect of Orientalist representations of the non-Western Other); the colonial difference (the epistemic division of modernity from coloniality and its use to create further divisions and differences in knowledge); and imperial difference (the downgrading and hierarchization of European others, for example, the Ottomans, the Chinese or the Russians) to assess and examine the constitution of the “Other” in European and North American fields of knowledge-in social sciences but also art, literature, and aesthetics. It argues for a need to excavate alternate epistemologies. Thus, the use of the term decoloniality is a perspective that is not tainted by Enlightenment thought. In this formulation, its key theorist, Mignolo (2007, 2017), suggests decolonial scholars can articulate their novel perspectives by using the methods of border epistemology, de-linking, and pluriversality to create alternate knowledge.^25^

## Anti-colonial social theory: Issues for further discussions

As mentioned earlier, global engagement with anti-colonial/imperial social theory is a recent development. In addition, much of this discussion globally has been restricted to post-colonial and decolonial perspectives.^26^ As against this, I have argued above that there are more versions of this perspective, that these have a long history stretching back to the late 19th century, and that its many versions could be divided into two phases. Furthermore, I have distinguished between five contemporary trends bringing these into conversation with each other. In order to push this discussion further, I suggest that it is imperative to ask two questions: (a) what new perspective(s) regarding the ontological–epistemological do these offer to the social sciences and (b) what methodological and theoretical issues do this short survey of anti-colonial social theory raise.

The first query relates to strategies presented by these five perspectives to comprehend the ontological-epistemological. Despite significant differences, the above-mentioned theories suggest a need to affirm a methodological assumption first articulated in anti-colonial movements that the knowledge of the “social” is ideologically associated with the processes of colonial-capitalism and that these represent global dominant/hegemonic perspectives. Consequently, it is argued that contemporary social science theories need to be mediated and filtered through a theory of politics of knowledge production. Understanding colonial/imperial geopolitics is a prerequisite to assessing both the theory of politics of knowledge production and modernity. The contemporary critique of Eurocentrism is a starting point for building an ontology from an anti-colonial perspective. This implies, first, a recognition of the power equation within the Eurocentric binary of the “I” and the “other.” Anti-colonial scholarship outlines ways to subvert it and find a new epistemic voice to define the “I.” This has led scholars to devise new methods to examine the politics of power: endogeneity in the case of Paulin Hountondji, structuralist deconstruction of the archive in the case of Ranajit Guha, and Marxist historiography in the case of Anibal Quijano. This search has also led to the analysis of the impact of colonized power on the constitution of hierarchies within colonized territories. This can be seen in Guha's distinction between the nationalist elite and the subalterns and Quijano's understanding of exploitation being organized in terms of both class and race. Second, these perspectives uphold a shift away from the linear theory of time/history and its theories of evolutionism. With colonialism, it is argued that there occurs an epistemic break, and history needs to start from there. Consequently, most anti-colonial theories of modernity enunciated within colonized regions assess the colonial/imperial spatial connections that organize the flows of commodities, ideas and ideologies, and fields of knowledge between metropole(s), semi-peripheries, and peripheries of the world.

More particularly, the contemporary anti-colonial social theory uses a combination of methodological strategies ranging from structuralism, post-structuralism, deconstruction with dependency theory, world system analysis and a critical Marxist historical sociology to interrogate Eurocentrism's attributes. Consequently, these have argued variously that dominant/hegemonic social sciences are (a) ethnocentric-these project a superiority of European experience of modernity, (b) that these universalize European historical and cultural patterns of modernity and thus promote path dependency, (c) sometimes reconstruct partially and sometimes efface non-European history in order to reproduce it through binaries that include racist, caste-ist, gendered and other categories of hierarchies, (d) divide and create boundaries and borders between social sciences, and (e) promote an Orientalist way to look at the non-European world.

However, in spite of this commonality, there is tenuousness in these discussions and sometimes where there is a conversation between them, we can note a tension concerning strategies to be framed for assessing dominant/hegemonic knowledge. This tension is best seen in the commentaries by decolonial scholars against post-colonialism, a discussion that continues to resonate today.^27^ The former argues that since the 16th century, Eurocentric assumptions were not only coproduced within Europe and Latin America but spanned across the world through migration, slavery, and communicative media. Thus, these found legitimacy and defined the proto-history of Western capitalist ideologies and, in turn, impacted knowledge fields as capitalism spread across the world. The decolonialists would contend that post-colonial critiques originating in 18th-century anti-colonial thought remain embedded in Western experiences of modernity and incorporates a Eurocentric bias. Instead, the decolonial perspective searches for a new epistemic voice outside western ideologies, which they suggest can be located in the experiences of the original inhabitants, or the indigenous groups in the regions of the Caribbean, Mesoamerica, and the Andes (Mignolo, 2017).

Despite these articulated differences, I would contend that these two perspectives have more in common in their styles of argument, even though they highlight their difference in terms of content.^28^ Both clearly distinguish themselves from Marxist scholarship (through different logics) and employ methodologies in use within humanities to assess contemporary discursive fields. Thus, while Edward Said mentions Marx's articles on India in the *New York Daily Tribune* and argues that these use orientalist sources and are embedded in the Orientalist perspective, Walter Mignolo suggests that there is a need to displace the entire Enlightenment thought, which includes Marx in order to search for alternative tools and theories in pre-Enlightenment perspectives.^29^ In addition, post-colonialism and decoloniality hardly ever engage with analysis and data that organize the field of political economy. Furthermore, they have tended to distance themselves from the diachronic perspective (for decoloniality, this is true as practice more than perspective). In addition, both tend to be anti-foundationalists. This attribute raises problems for social sciences, for it becomes difficult to study the “real world” if “true” descriptions are completely rejected (Patel, 2021b). Finally, both seem to argue that doing post-colonial and decolonial interventions implies doing praxiological work.

In addition to the distinction between post-colonial and decolonial, there is within American academia a domain called Empire studies. Mainly associated with George Steinmetz, it focuses on sociology as a discipline and examines how German and British sociological scholarship was imbricated in the colonial project. In his 2016 text titled *Post-colonial Thought and Social Theory*, Julian Go also uses the theory of empire studies and relates it to post-colonialism. In this book, he focuses on reconstituting sociological theory through network theory. Recently Go (2023) used empire studies to integrate it with anti-colonial thought and suggests that anti-colonial thought has a built-in anti-colonial social theory, which he identifies in terms of three attributes: the self, solidarity, and the global. In this discussion, he brings a range of thinkers from the Philippines, such as Mabini or Kellog from the First Nations, the sociologist Dubois, Frantz Fannon, Amilcar Cabral from the Caribbean, Nyugen An Ninh from Vietnam, and Radhakamal Mukerjee from India. Go, in my opinion, is discussing anti-colonial thought, which he equates with anti-colonial social theory. My position on social theory is different than his-as. I distinguish between anti-colonial thought and anti-colonial social theory. Anti-colonial thought, by definition, is interdisciplinary, while anti-colonial social theory is meta-theory, and as outlined earlier, it is an exploration of the ontological–epistemological.

These discussions raise another issue: can one and should one distinguish between two different kinds of anti-colonial thought; the first that derives from examining the experiences of slavery and migration and the one that discusses the exploitation of people and territories. This is about settled and non-settled colonialism. The former has yielded an efflorescence of ideas and is known as black/radical African thought. It integrates African, African American, Afro-American, Afro-Asian, Afro-European, Afro-Latino, Afro-Native American, Caribbean, Pan-African, and Black British thought in its perspective (Rebaka, 2009). Here, the discussions focus on the colonization of bodies rather than territories and move into cultural studies. And though Fanon, Cabral, and Cesare are appropriate in these discussions, the latter is associated with the second kind of anti-colonial thought. These debates suggest a need to clarify further the theoretical framing of social theory and anti-colonial thought.

This brings me to the second point: the issues that need further discussion. While today colonialism is being used as a paradigmatic concept, it is necessary to distinguish between various kinds of colonialism. Early literature on this theme highlighted the differences between English, French, Dutch, and German colonialisms. Mignolo (2005), as mentioned earlier, has also made a sharp distinction between the meta-theoretical issues raised by the post-colonial scholarship located in British colonialism of the 18th century and those raised by Quijano in his assessment of Iberian colonialism. Recent work by historians has suggested a need to distinguish between the way colonialism has impacted settled and non-settled colonialism. These have gestured for a need to comprehend need to distinguish the very long history of Iberian colonialism from British colonialism in order to comprehend the distinct articulations of colonialism/imperialism in its institutions, ideologies, and practices.^30^

Social scientists studying settled colonialism have argued that successful settler colonies had tamed the original inhabitants considered “wild” through Christian religiosities. Only recently has this repression and co-option, which sometimes have led to the complete extinction of original inhabitants through genocide, been theorized in comparative terms within regions and across continents where settled colonialism was practiced. Certainly, racialization influenced by Christianity was the key to this process of domination within parts of settled colonial territories. However, it is now recognized that it was formulated in distinct ways in various regions of settler colonialism as these interfaced with pre-colonial institutions and practices. From these engagements emerged state bureaucracies, legal mechanisms, and ideologies that helped to legitimize colonialism differently in various regions and sub-regions across continents. In addition, historians have argued that as colonialism spanned over 400 years in new ways, modernity imposed by settled colonialism within various regions evolved in new directions and led to further distinctions within and between these regions.^31^ In non-settled colonialism, a sustained drive for material exploitation led by the native elite at the behest of the colonial elite has led to the subordination of the colonized through the reconstitution of local hierarchical systems, such as caste, in the case of India. In some of these regions, pre-modern theories of difference and hierarchy have legitimized the colonial-capitalist processes. Obviously, further research needs to be done to assess these trends within various regions and across various continents of the world, together with the assessment of the use of the same concepts with different meanings used by scholars in these two regions^32^ (Patel, 2021b).

Second, can these toolkits be used in comprehending the “social” in territories and situations where colonialism has not left an imprint? Or, to put it differently, does the colonial discourse stamp itself in territories which has not experienced colonialism? Recent literature on eastern regions of Europe and Russia and the “Far East” have argued that the assumptions defining coloniality have also impacted social sciences in these regions. Earlier, Fernando (1996) argued that forms of Occidentalism had impacted the reproduction of knowledge in various parts of Europe. Scholars have explored methods needed to assess and reconstruct social sciences in territories that were not part of the capitalist colonial/imperial system, such as Russia and eastern parts of Europe but had their own set of (socialist) imperialist premises (Tlostanova, 2012). Discussions have also assessed the social sciences in Japan, which was colonized at one point but had also colonialized Korea. Recently, the historian of the “Far East,” Barlow (2012), has argued that we need a new perspective, which she calls a theory of colonial modernity, to substitute theories of modernization and transnationalism, which use static categories such as nation, modernity, tradition, culture, stages of development and civilization with those that assess multiple and overlapping projects of contemporary colonialism and imperialisms. She suggests that today the circulation of commodities across the world has established and reshaped styles of governmentality, juridical norms, administrative innovations, and intellectual discourses, thereby legitimizing domination-subordination structures defining the metropole(s) with the peripheries actualized first during colonialism. She contends that these flows define all spaces of everyday life, even those which have not experienced colonialism. This position has found support among scholars who work in China, Japan, Korea and Thailand, Cambodia, and Hong Kong (Mackie, 2018).

Third, contemporary debates suggest a need to comprehend the role played by nationalism, the nation-state, its institutions, and the nationalist elite in promoting the Eurocentric episteme. It was first mentioned by the subaltern theorist Guha (1982), who criticized nationalist historiography for its European understanding of the political domain and consequently presented subaltern consciousness in all its complexity as an example of an alternative way of thinking about modernity. Recently, drawing from Partha Chatterjee's theory of derivative nationalism, Patel (2017) has contended that the two assumptions of Eurocentrism-linearity and binaries-embedded themselves within Indian nationalist social sciences as these engaged with western social sciences in India. The latter were divided into traditional and modern social sciences or those dealing with the arenas of the private and public domains, that is, sociology and anthropology as against economics and politics. Sociology and anthropology visualized the private domain as steeped in traditional institutions- the extended family, caste, and Hindu religion, while economics and politics reproduced Western liberal conceptual understanding of the state and market. Thus, she argues that after the independence of India, methodological nationalism reconstituted the epistemic assumptions of Eurocentrism.^33^ This perspective begs the question: In what way have nationalism and nationalist assumptions implicated the framing of social sciences in other regions of the world?

Fourth, it is necessary to situate and place anti-colonial social theory in terms of current global geopolitics and the organization of contemporary knowledge systems and their institutionalized flows of ideas. The anti-colonial social theory was the first to discuss the existence of colonized circulation of knowledge fields connecting the peripheries–semi-peripheries–peripheries. It has also queried the discourses that determine who produces knowledge for whom and its reproduction through its global circulation. Anti-colonial social theorists have suggested that these divisions create multi-scaler geographies of knowledge flows, and contemporary critiques, such as the UNESCO report of 2010, titled *Knowledge Divides*^34^ confirms this trend. Some anti-colonial theorists have argued that these divisions have materialized despite huge investments by ex-colonial nation-states to create an intellectual infrastructure to develop autonomous social sciences.^35^

Paulin Hountondji has highlighted the problem as perceived from the experience of African nation-states. Hountondji uses his theory of extraversion (externally produced knowledge) to assess the cultural and institutional structures that organize academic dependencies. He argues that if African social sciences lack the autonomy to develop scientific practices to re-invent themselves, this is so because they lack financial and human resources, have a small number of universities and research institutes, and in the publishing industry, they have little to no physical infrastructure to house libraries and archives; scholars lack intellectual confidence to promote research questions, themes and specializations that are relevant to their own peoples. In addition, Hountondji argues that an absence of the philosophical location of concepts and their scientific understandings fuels and promotes academic tourist circuits with diasporic scholars who are influenced by the metropolitan research questions and their methods.^36^

The dependencies that Hountondji has outlined are related to the continuing inequality of global knowledge production. This is associated with an investment in educational institutions, such as universities, research institutions, and laboratories, as well as in the publishing industry. Related to this is the universalization of practices regarding teaching, learning, and pedagogy, curriculum and syllabi, books and review protocols, and Eurocentric disciplinary divides fashioned by knowledge fields in core capitalist countries. However, evidence suggests that there was a backward flow of subaltern knowledge from colonizing regions to colonial countries and attempts made for horizontal linkages across the global south in the late 50s through the Bandung initiative and since the early 21st century through BRICS, the divisions structuring knowledge flows have not been diminished. Rather, these have become enhanced with the use of new media technologies. Thus, the question: In what way can anti-colonial social theory intervene in the existing divisions of knowledge that organize knowledge around the globe?

## Conclusion

The article maps the variety of trends associated with anti-colonial social theory and discusses its two phases since its emergence in the late 1940s. It suggests that contemporary sociological scholarship has to move beyond post-colonial and decolonial approaches to comprehend this variety and its contributions. It also suggests that these various trends are bound together by a common ontological–epistemological perspective, and despite the differences between these perspectives, these raise fundamental queries regarding the way theory of theory needs to be conceptualized.

The study suggests that the anti-colonial social theory affirms the need to map context, time, and space to organize research queries and methods and to comprehend the processes, mechanisms, and events that impact action and actors in colonized and colonizing worlds. This social theory helps to examine how to build substantive theories on modernity, confirm its relevance, investigate empirical data, and apply it to carry out an empirical investigation. In order to understand this relationship between ontology and epistemology, the article presents the different strategies outlined by varieties of anti-colonial social theory. It is also argued that anti-colonial social theory also demands an investigation of its own historical legacies, its institutional structures, and the changes that it has been able to incorporate to do a theory of theory.

One of the anti-colonial social theory's major contributions is assessing pathways structuring cognitive geographical circuits organizing the metropoles, their semi-peripheries and, in turn, the continuously expanding peripheries of the semi-peripheries. It argues that these divisions have created knowledge territories and borders of debate and deliberation and have been sustained by divided academic language communities. These multiple and overlapping knowledge projects connect the global, regional, national, and local academic communities and simultaneously reflect how these circuits reproduce themselves across various scales in unequal and uneven discourses of modernities. The many circuits that one can identify and the many terms it has used to self-define itself suggest a need to comprehend the diversities that organize anti-colonial social theory. While acknowledging that geopolitics continues to organize flows of knowledge, anti-colonial social theory, the article argues, is best equipped to intervene in these geopolitics of knowledge production and circulation.

Anti-colonial social theory has presented ways to deconstruct these institutionalized flows of knowledge circulation and reproduction. Given this, its discussions help to reframe global social theory's fragmented field by asserting that these differences do not indicate a closure of the field. Rather these constitute an opportunity to understand the necessity for social theory to accept diversities organized by colonialist and imperialist spatial flows. A universal theory, a “one fits all” position, has become dysfunctional. This means that scholars need to write histories and sociologies examining the circulatory connections organized in differing scales and knowledge practices as these flow across cores, semi-peripheries, and peripheries. The above discussion makes it obvious that a significant amount of scholarly work must be done to clarify how colonial knowledge continues to be reproduced even as efforts are made to map and intervene to disrupt its dominant trends.

We live in an interconnected global world. In the 21st century, we are integrated by many complex cognitive circuits within the overall divisions created by its colonial and nationalist histories, geographies, and the unequal distribution of income, privilege, status, and power. These circuits have created differences and variations within the global world system consequent to the way events and processes have been organized, and sociabilities constituted. Subsequently, it is imperative to understand these cognitive geographies and the system that reproduces them and to do research through these varying scales, bringing back local and regional scholarship within the global as it connects the scholarship of organic intellectuals with formal knowledge. Anti-colonial social theory has seen this as its most important task and thus its relevance as a global social theory.

## Data availability statement

The raw data supporting the conclusions of this article will be made available by the authors, without undue reservation.

## Author contributions

The author confirms being the sole contributor of this work and has approved it for publication.

## Footnotes

^1^In this context, it would be apposite also to note the discussions on “internal colonization” (Martin, 2018).

^2^Lindner (2022) contended that Marx's writings on Russia and Ireland, published in the comprehensive edition of his study, suggest that he had displaced his earlier Orientalist assessments found in his 1850s writings on India and published in the *New York Daily Tribune*. This intervention is to counter postcolonial perspectives on this issue.

^3^Some trends within anti-colonial thought, in particular within postcolonial and decolonial scholarship, set themselves apart from Marxist perspectives.

^4^In this study, the two terms are used interchangeably.

^5^Given the uneven space-time moorings of anti-colonial thought, it is difficult to compress its emergence and spread in linear time frames.

^6^For example, economic nationalism in India in the late 18^th^ century; Gandhi's anti-industrialization philosophy in his book *Hind Swaraj*; anti-imperialist dependency theory of Lain America or the theory of racist colonialism of the Atlantic-African perspective; or of indigenous perspective promoted in northern Africa. These have had many multi-linear origins and growth.

^7^Go (2022) outlined three problematics of anti-colonial thought. These are as follows: an assessment of self and society; an understanding of social solidarity; and a theorization of inter-societal connectedness, global hierarchies, and the constitutive character of the empire.

^8^In South Asia, this has led to the growth of the Ambedkerite movement, anti-caste movement, and a civil society initiative against communalization (religious divides) of society. For a similar process in Africa, see Mamdani (1996).

^9^This could be the reframing of caste, ethnicity, tribal, ethnic, and religious boundaries and segmenting these.

^10^Post-colonialism can be defined “as that branch of contemporary theory that investigates, and develops propositions about, the cultural and political impact of European conquest upon colonized societies, and the nature of those societies' responses” (Ashcroft, 2012, p. xv). Leela Gandhi suggests that “post-colonialism can be seen as a theoretical resistance to the mystifying amnesia of the colonial aftermath. It is a disciplinary project devoted to the academic task of revisiting, remembering and, crucially, interrogating the colonial past. The process of returning to the colonial scene discloses a relationship of reciprocal antagonism and desire between colonizer and colonized. And it is in the unfolding of this troubled and troubling relationship that we might start to discern the ambivalent prehistory of the postcolonial condition” (Gandhi, 1998, p. 4).

^11^Mignolo (2017) defined decoloniality as a means first to delink (to detach) from that overall structure of knowledge in order to engage in an epistemic reconstitution. Reconstitution of what? Of ways of thinking, languages, ways of life, and being in the world where the rhetoric of modernity is disavowed and the logic of coloniality implemented.

^12^Connell (2014) argued that there are four kinds of intellectual projects within Southern theory: (a) preservation of indigenous knowledge and practices; (b) thinking about the invasion; (c) understanding the new society created by colonialism and neo-colonialism; and (d) assessing knowledge and reconstructing it in new ways.

^13^These are background assumptions that operate in the shadows and determine research questions and methods to be used and influence the framing of concepts and logics of inquiry and legitimize how events and processes are related to observed data and experiences and thus justify explanations. These assumptions are philosophical, sociological, and psychological (see also text footnote ^9^).

^14^For Bourdieu, the interrogation of the “collective scientific unconsciousness” implies an examination of its embedded nature “in theories, problems, and (especially national) categories of scholarly judgement.” This should be the subject of inquiry and of reflection because, for Bourdieu, it is social science itself that should be made the object of investigation (Wacquant, 1992, p. 40).

^15^No wonder Indian anti-colonial thought and its subsequent analytical stance made a major contribution tp the field of developmental economics.

^16^I have explored the debates around indigenous thought in the context of two theorists: Akiwowo Akinsola and D. P. Mukerji (Patel, 2021a,b).

^17^A detailed review of the indigenous traditions is available in Patel (2015) and Patel (2021a,b).

^18^In recent times, black radical/Africana thought, also called Atlantic-African thought, has been called anti-colonial thought because of its engagement with African, African American, Afro-American, Afro-Asian, Afro-European, Afro-Latino, Afro-Native American, Caribbean, Pan-African, and Black British thought (Rebaka, 2009; Go, 2022). It has been reconstituted through an engagement with the scholarship of W. E. B. Du Bois, C. L. R. James, Aime Cesaire, Leopold Senghor, Frantz Fanon, and Amilcar Cabral. However, with the exception of Fanon, who critically dissects bourgeois nationalist capitalism, Africana thought does not deal with colonizing of territories and their peoples. Thus, it is not engaged with here. See note 4.

^19^In this article, I have restricted the discussion to the African use of the method of indigenous. However, there are many other variants of the same. In this context, see Smith (1999) and Alatas (1972, 2006).

^20^A recent book on Paulin Hountondji examines his methodological interventions ideas in detail (Dübgen and Skupien, 2019).

^21^Ludden (2002) brings together a series of assessments on the subaltern studies project which raises methodological issues that confront this scholarship.

^22^There is an extensive critical assessment of postcolonialism. See Schwarz and Ray (2005), and a recent work is that of Albrecht (2020). For a sociological critique, see Turner (1989).

^23^The decolonial perspective moors the framing of its anti-colonial thinking in Anibal Quijano's study. However, in this study, I distinguish between the latter and decolonial perspective, given Quijano's very clear association with Marxist historiography.

^24^A critical assessment of Anibal Quijano's contribution is available in Gandrilla et al. (2021).

^25^See Gopal's (2022) methodological criticism of Mignolo's perspective on decoloniality.

^26^That the discussion remains limited to post-colonialism and decoloniality is evident in Bhambra (2014b). Tilly (2017) has cautioned against the reproduction of post-colonial/decolonial perspectives as dominant/hegemonic positions. She suggests a need to assess the political economy of knowledge production—its forms of extraction, points of commodification, how it is refined as intellectual property, and how it comes to alienate participating knowers.

^27^Recent issues of the journal *Postcolonial Studies* and *Hypatia* deliberate on these conflicts. See Asher and Ramamurthy (2020) and Colpani et al. (2022).

^28^For a different point of view, see Davis and Walsh (2020).

^29^See text footnote ^2^. Lindner (2022, p. 7) also argued that this was recognized by Indian Marxist historians, such as Bipan Chandra.

^30^The publication of a new journal on settler colonialism establishes a need to separate the two kinds of colonialism and its knowledge domains (Veracini, 2011).

^31^The historian Ricardo D. Salvatore has elaborated how spatial distinctions emerged within Latin America as different institutions and practices found expression in the continent (Salvatore, 2010).

^32^Of particular importance is understanding the distinct usages of the term “indigenous” within settled and non-settled colonialisms. Moreover, there is the need to distinguish between the way Walter Mignolo and Partha Chatterjee define “colonial difference.”

^33^Frantz Fanon, in his essay on “The Pitfalls of National Consciousness” in *The Wretched of the Earth*, also raises similar problems with regard to nationalism. See Lazarus (1993).

^34^It contends that in spite of some progress, the production of social science knowledge remains concentrated in North America and Europe and that social sciences are practiced mainly in the English language.

^35^See, in this context, the experience of Nigeria as illustrated by Onwuzuruigbo (2018).

^36^Alatas (1972) makes a similar argument.
